# Supplementary figures and images for: Anti-methicillin-resistant Staphylococcus aureus and antibiofilm activity of new peptides produced by a Brevibacillus strain
Source: PeerJ. 2023 Oct 2;11:e16143. doi: 10.7717/peerj.16143 (PMC10552749; doi:10.7717/peerj.16143)

**A. BrSPR19-P1**

**
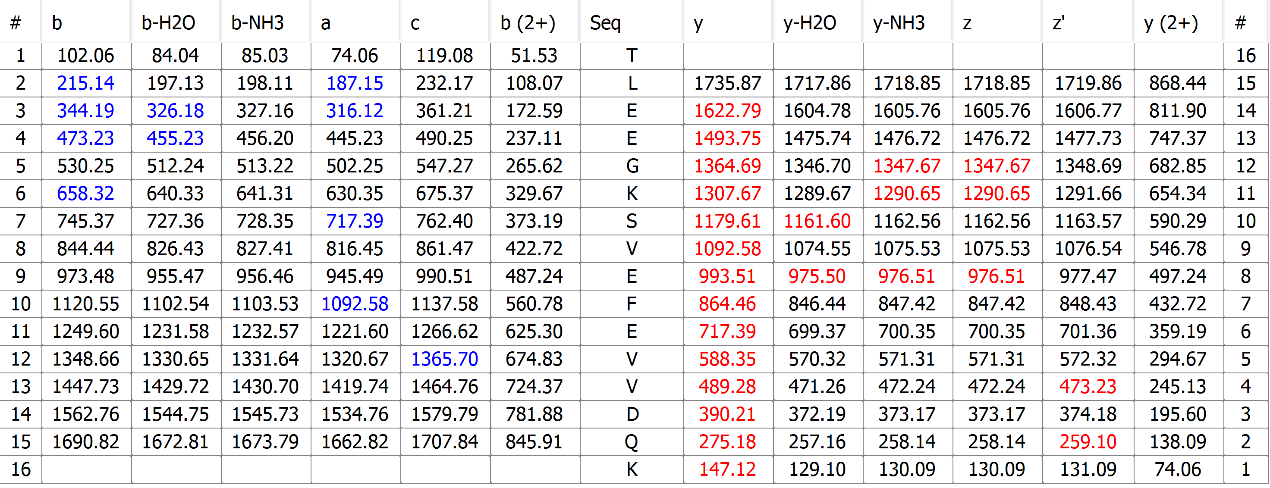
**

**B. BrSPR19-P2**

**
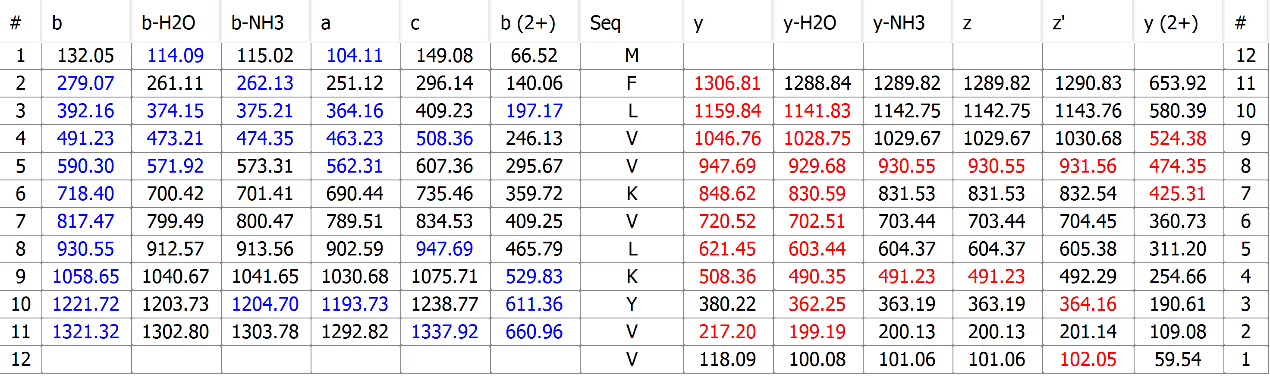
**

**C. BrSPR19-P3**

**
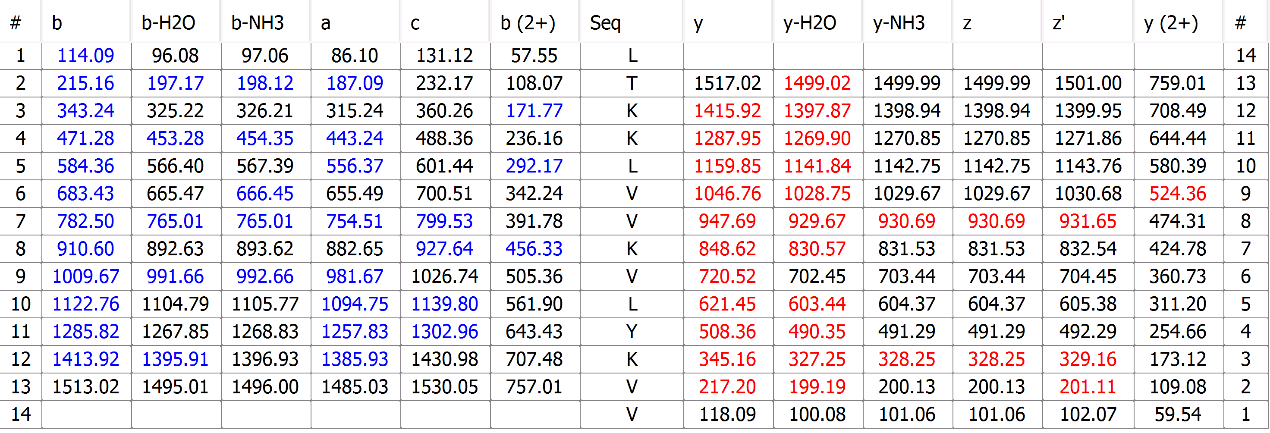
**

**D. BrSPR19-P4**

**
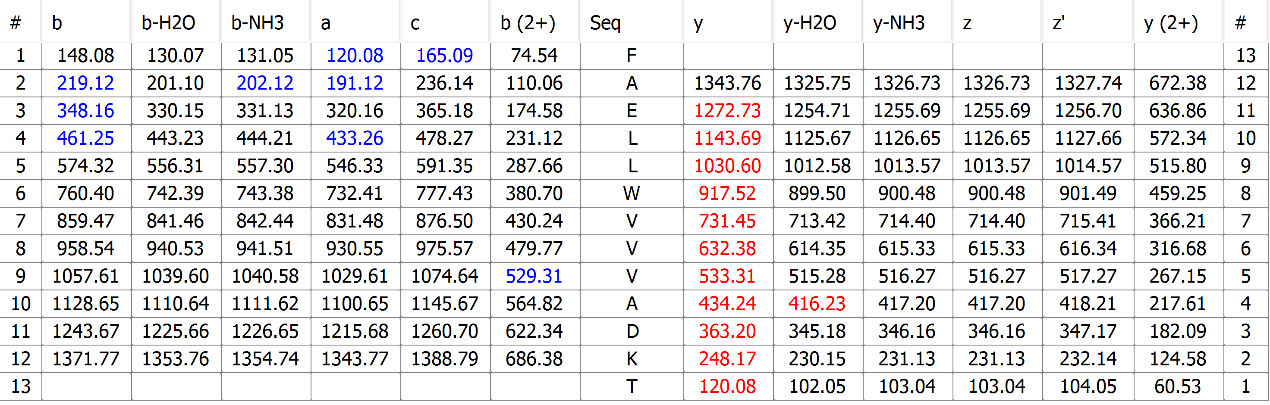
**

**E. BrSPR19-P5**

**
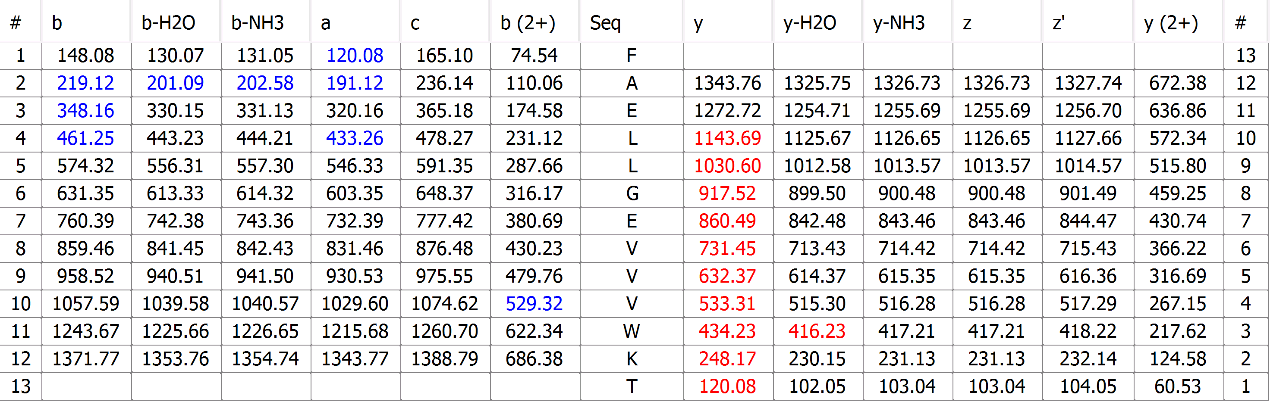
**

Supplement: Supplemental Information 1 — (A–E) BrSPR19-P1 to BrSPR19-P5. [file peerj-11-16143-s001.docx]

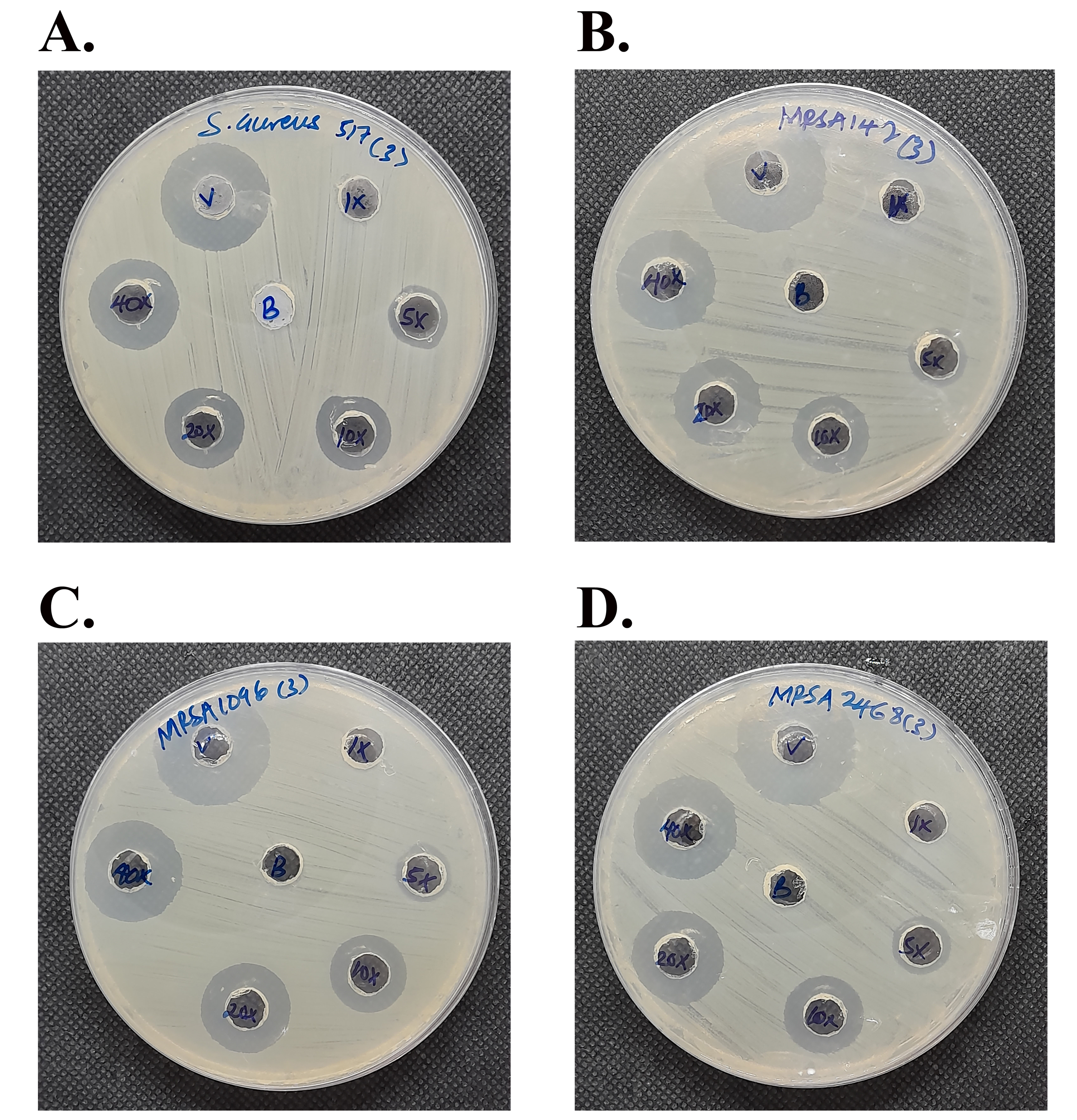

Supplement: Supplemental Information 2 — The P2 peptide at the different concentrations (1×, 5×, 10×, 20×, and 40× MIC) exhibited inhibition zones against (A) S. aureus TISTR 517 and (B–D) MRSA isolate 142, 1096, and 2468, respectively. Vancomycin (30 μg or 150′ MIC) was used as a control drug. [file peerj-11-16143-s002.png]

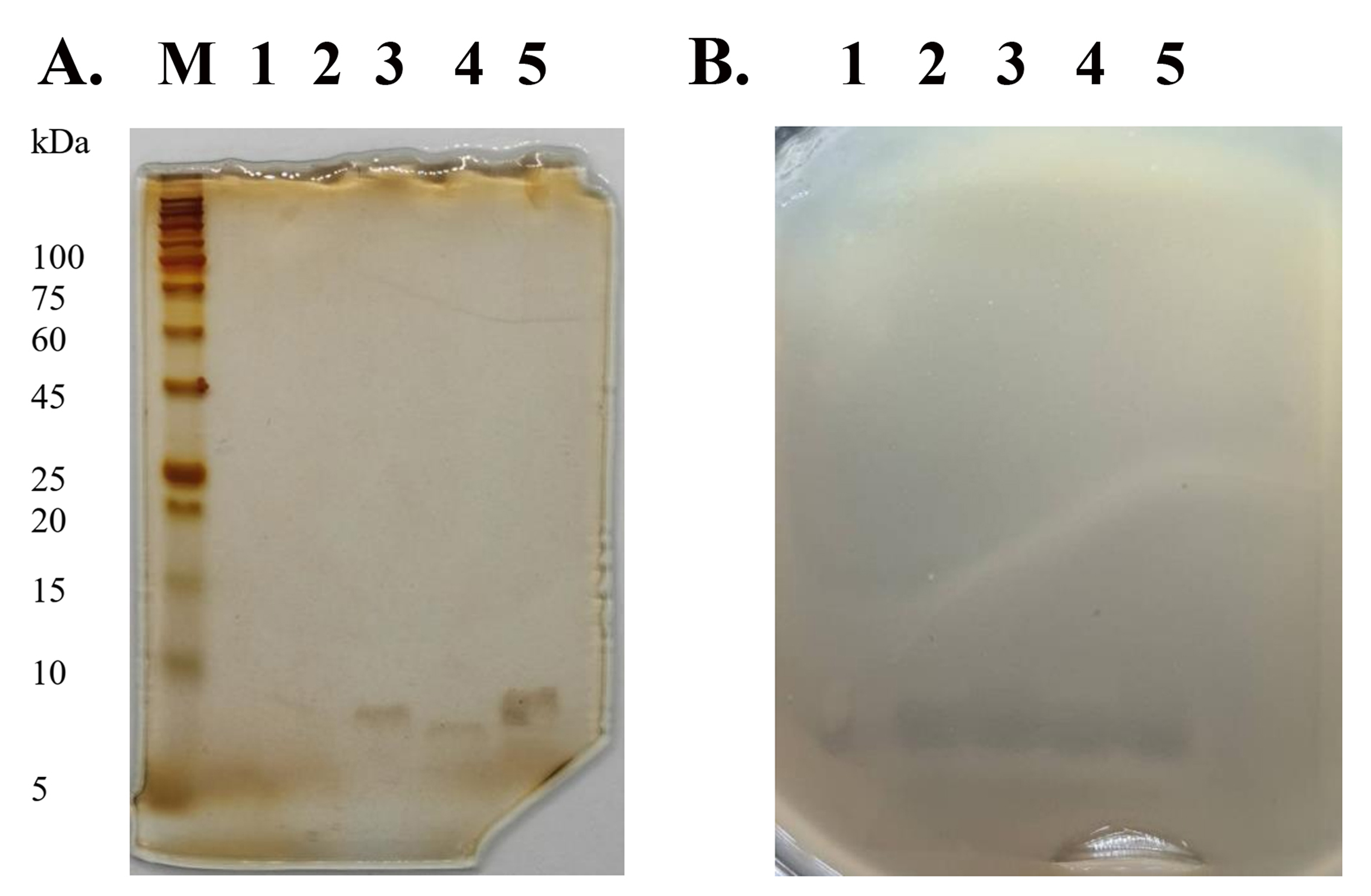

Supplement: Supplemental Information 3 — (A) Silver-stained SDS-PAGE gel of peptides from SPR19. Lane M, protein marker; Lanes 1−5, P1−P5, respectively (B) Agar overlay assay with MRSA isolate 2468. The corresponding gel was overlaid by soft agar, containing the tested bacteria. The plate was incubated at 37 °C for 24 h. [file peerj-11-16143-s003.png]

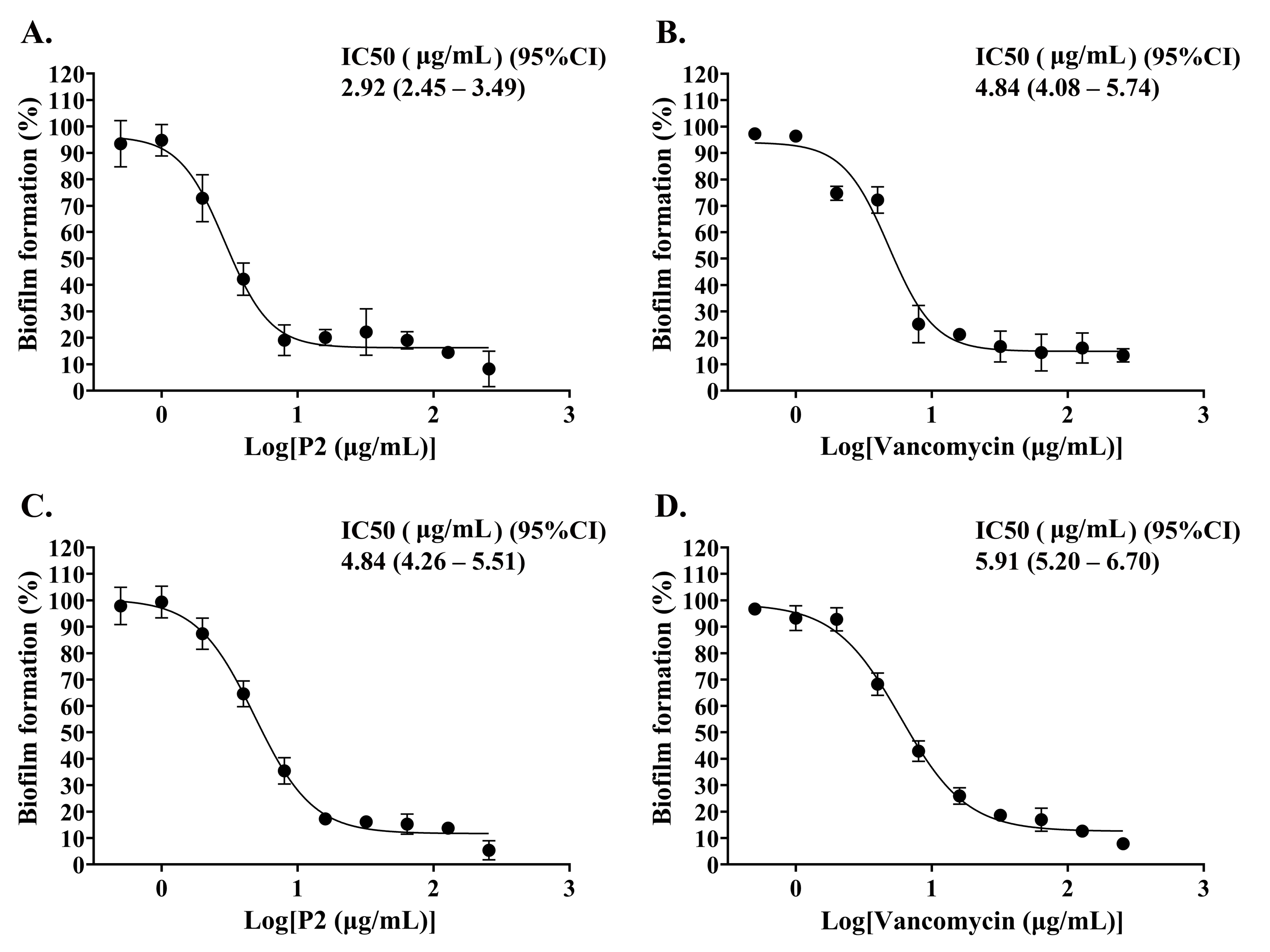

Supplement: Supplemental Information 4 — (A and B) S. aureus TISTR 517 and (C and D) MRSA isolate 2468 were treated with P2 and vancomycin, respectively, and the biofilm production was measured. The Hill model was used to calculate the concentration of tested substances that inhibited biofilm formation by 50% (IC50). [file peerj-11-16143-s004.png]
